# Isotretinoin; A review on the Utilization Pattern in Pregnancy

**DOI:** 10.15171/apb.2018.044

**Published:** 2018-08-29

**Authors:** Sajad Khiali, Afshin Gharekhani, Taher Entezari-Maleki

**Affiliations:** ^1^Department of Clinical Pharmacy, Faculty of Pharmacy, Tabriz University of Medical Sciences, Tabriz, Iran.; ^2^Drug Applied Research Center, Tabriz University of Medical Sciences, Tabriz, Iran.

**Keywords:** Isotretinoin, Acne, Utilization pattern, Teratogenic effects, Pregnancy

## Abstract

***Purpose:*** Isotretinoin is the most effective anti-acne drug with a long-term remission. However; it contains severe teratogenic effects with serious adverse drug reactions, which limits the use of medication.

***Methods:*** To review the use of isotretinoin during pregnancy, we carried out a comprehensive search of literature in Google Scholar, Scopus and PubMed/Medline from their inception until April 2015.

***Results:*** Database searching identiﬁed 277 records, of which, 38 articles were retrieved according to abstract and title assessment. After full-text review, 17 articles were excluded and finally, a total of 21 studies met the inclusion criteria. Data showed an increased pattern in the use of isotretinoin. In some studies, health care providers were not fully adhered to the risk reduction programs in pregnancy. Exposing to isotretinoin among pregnant women has still occurred due to detrimental adherence to risk reduction programs which resulted in live-born infants with different kinds of abnormalities.

***Conclusion:*** Despite the known serious adverse effect of isotretinoin, the use of drug was not based on the guidelines in some cases, which needs more attentions to prevent the severe drug related problems.

## Introduction

Acne vulgaris, or simply acne is the most common disorder of skin that affects the majority of young adults.^1,2^ It creates undesirable appearance with subsequent difficulties including depression and adverse effects on body image, self-stem, and quality of life.^3-6^

Several medications have been approved for the treatment of acne, but oral isotretinoin (13-cis retinoic acid) is the most clinically effective anti-acne therapy, producing long-term remission of acne symptoms by improving all the factors responsible for the generation of the disease. Based on published studies, oral isotretinoin is the gold standard treatment for serve nodulocyctic and recalcitrant acne.^7^ Most studies have suggested a cumulative dose of 120 to 150 mg/kg to reduce the risk of disease relapse.^8-12^ However, some newer studies found a lower rate of relapse with high-dose oral isotretinoin while others reported a comparable response rate with lower side effects using low-dose isotretinoin.^13-18^ despite the well efficacy of isotretinoin in the acne therapy, several serious adverse effects including pancreatitis, depression, and suicidal thoughts have been reported. Most importantly, the teratogenicity is the major limiting factor for systemic isotretinoin use.^19-23^

Two nuclear ligand-induced receptors (retinoic acid receptors and retinoid X receptors) seems to have important role in retinoid teratogenicity through other downstream important genes in development. It has been suggested Hox gene expression causes the harmful effects of retinoic acid on the development of structures from the neural crest derivatives.^24,25^

The literature review indicated that isotretinoin use and prescription have been increasing all over the world.^26-33^ For instance, a 2.5-fold increase in the use of isotretinoin was showed in the US between 1992 and 2000.^31^ The Ministry of Health and Education of Iran reported a 4-fold increase in the use of isotretinoin between 2000 and 2007.^32^ The well treatment efficacy of isotretinoin in the management of acne besides a higher failure rate and improper use of other therapeutic modalities may have contributed to the increased use of oral isotretinoin.^33^ Due to the considerable increase in the use of isotretinoin along with its serious adverse effects on pregnancy, this study was performed to evaluate the utilization pattern of isotretinoin in pregnant women.

## Methods

### Data Sources and Search Strategy

A systematic search of literature was carried out in 3 major databases including PubMed/Medline, Scopus and Google Scholar from January 1980 up to April 2015. The keywords used as search terms in databases included "isotretinoin" and "pregnancy" and "prescription / use / utilization "and "isotretinoin" and "prescription / use / utilization". Reference of retrieved articles were also screened for inclusion of any additional related studies. Furthermore, a comprehensive searching of Cochornce, Science Citation Index, Embas and ISI Web of Knowledge was carried out. It is revealed that there were no additional studies.

### Inclusion and Exclusion Criteria

All studies on isotretinoin concerns, awareness and its utilization in the treatment of acne were investigated. Articles published in Non-English languages, describing acne pathology or its adjuvant care as well as without full text availability were excluded.

## Results and Discussion

### The utilization pattern

Searching in databases identiﬁed 277 records, of which, 38 were relevant according to their title and abstract. After full-text review, 17 articles were excluded because they were irrelevant (n=5) or had no available free full-text (n=12). Finally, a total of 21 studies met the inclusion criteria.

There are a limited number of studies reporting utilization pattern of isotretinoin in the world. For example, one study in the US showed a 2.5-fold increase between 1992 and 2000.^31^ Furthermore, a dramatic increase of 4-fold in the use of isotretinoin from 2000 to 2006 has been reported in developing countries such as Iran.^32^

In addition to increased use and prescription of isotretinoin, the improper use of the drug was reported in some studies. According to a study of Wysowski DK in the USA, isotretinoin rate of prescription for severe acne declined from 63% to 46%, whereas this rate for treatment of mild and moderate acne increased from 31% to 49% between 1993 and 2000.^31^ Similarly, another prospective study on 274 Iranian patients indicated the improper prescriptions of isotretinoin for mild and moderate acne in 20% of patients. In this study, isotretinoin was prescribed under the usual recommended daily dose of 0.5-1 mg/kg in 51.3% of cases. Furthermore, 12.2% of cases received beyond cumulative dose of 150mg/kg and 33.5% of them were treated with less than cumulative dose of 120mg/kg.^26^ In addition, based on data of Korean nationwide study, it was demonstrated that most of the physicians prescribed isotretinoin as the first-line therapy for the treatment of moderate acne.^34^ A retrospective study in Dutch indicated that only 70% of patients were prescribed conventional acne therapy prior to receiving isotretinoin.^35^

### Use of isotretinoin and pregnancy consideration

Due to the teratogenic effects of isotretinoin, a series of risk reduction programs including Pregnancy Prevention Program (PPP), System to Manage Accutane Related Teratogenicity (SMART) and iPLEDGE (a mandatory distribution program in the United States for isotretinoin) have been established in order to inhibit pregnant women from taking the medication and to prevent women taking the drug to get pregnant.^36,37^

In spite of implementing the PPP and Dear Healthcare Professional Letter (DHPL), a descriptive study on first-time receivers of isotretinoin also demonstrated inappropriate use of isotretinoin.^38^ As well, SMART inadequate success in reducing isotretinoin-exposed pregnancies led to the development of iPLEDGE in the USA which is stricter than the other risk reduction programs.^38-42^

### Reports from Middle East

Considering the lack of risk reduction programs in Iran, a drug utilization evaluation (DUE) study on 274 outpatients treated with isotretinoin, demonstrated that only 6.8% of couples used two concomitant methods of contraception.^26^ Furthermore, according to a survey of 116 pharmacists’ knowledge on isotretinoin teratogenic effects in Saudi Arabia, it was illustrated that community pharmacists were not sufficiently aware of its risks in female patients. Unexpectedly, 20% of pharmacists dispensed isotretinoin without any prescription. In addition, only 6.2% of them recommended two methods of contraception for females with childbearing potential.^42^

### Reports from the US and North America

A study was carried out to appraise the patterns of isotretinoin use and assessed concomitant use of isotretinoin and contraceptives among females aged 13 to 45 years before and after iPLEDGE implementation between 2004 and 2008 in the USA. According to findings from Intercontinental Marketing Services (IMS) Health longitudinal prescription claims database, the number of isotretinoin prescriptions declined after iPLEDGE implementation. On the other hand, a small but significant increase in concomitant use of isotretinoin and contraceptives was observed immediately after iPlEDGE implementation. However, through the time, contraceptive utilization pattern did not significantly change before and after the implementation of iPLEDGE.^43^

A retrospective comparative study between iPLEDGE and SMART programs was done in a managed care setting. All females of childbearing potential at Kaiser Permanent Southern and Northern California with at least one prescription of isotretinoin from 1 March 2004 to 29 February 2008 were enrolled. A total of 9912 isotretinoin treatment courses and 29 fetal exposures to isotretinoin were reported. It was demonstrated that the risk of fetal exposure after iPLEDGE implementation decreased significantly in comparison with the SMART program.^44^ Furthermore, a prospective Canadian population-based study on 8609 females receiving isotretinoin therapy was carried out to assess the rate of pregnancies exposed to isotretinoin from 1984 to 2002. It was demonstrated that 90 patients became pregnant among those participations. Approximately 85% of patients (n=76) terminated the pregnancy, and three spontaneous abortions and two neonatal deaths occurred. Finally, nine women had a live birth.^45^

### Reports from the Europe

A systematic review of 17 studies evaluated the compliance rate with the PPP in the Europe. The findings showed that 0.2–1per 1000 women of reproduction age receiving isotretinoin became pregnant whereas 65% – 87% of these pregnancies were terminated. Furthermore, only 6–26% of prescriptions were in accordance with the PPP.^46^

According to a prospective study from 1993 to 2008 in Germany, a total of 108 pregnant women exposed to isotretinoin at the medium daily dose of 20 mg. In spite of running PPP, none of them used two complementary contraceptives. Among them, 39 pregnant women had no information about contraception usage during isotretinoin consumption. Among 69 alert subjects, contraceptives failed in 21 patients and 48 patients used no contraception methods or stopped their use too early. Approximately 81% of established pregnancies were terminated. On the other hand, one major birth defect was seen among 18 live births (with 1 pair of twins).^47^ Another study was carried out to evaluate all cases of exposure to isotretinoin during pregnancy who which was voluntarily reported to pharmacovigilance centers, the Teratogenic Agent Information Centre, and pharmaceutical companies in France and all cases who received isotretinoin during pregnancy from 1 January 2003 to 31 December 2006, were assessed. A total of 147 cases of isotretinoin exposure were reported. They divided into 3 groups; first (n=61) who get pregnant less than 1 month after isotretinoin discontinuation; second (n=23) who became pregnant during isotretinoin use and third (n=16) who were started isotretinoin while they were pregnant. Prescription of isotertinoin was done by dermatologists in 86% of cases. In addition, 19% of women did not use any contraception method.^48^

A total of 203962 pregnancies corresponding to 208161 fetuses were evaluated in a population-based study in the Netherlands between 1 January 1999 and 1 September 2007 to assess their exposure to isotretinoin. It was demonstrated that 51 women had exposure to isotretinoin. Six patients became pregnant during the last month of discontinuing isotretinoin and 45 patients exposed to isotretinoin during pregnancy. Among those potentially exposed to isotretinoin, three intrauterine deaths and two live-birth delivery with major congenital anomalies occurred.^49^ In addition, a descriptive study was carried out between 2007 and 2011 to evaluate the compliance of pharmacists and dermatologists with PPP in the Netherlands. The data indicated that only 64% of dermatologists always prescribed contraception. furthermore, 22% of them prescribed it occasionally. In addition, approximately 45% of pharmacists checked the use of contraception method and 15% of them asked for a negative pregnancy test. Furthermore, Pharmacists' checks on 30-day dispensing was 82% and didn’t change, whereas a check whether the prescription is out-of-date decreased from 61% to 53% from 2007 to 2011. In addition to poor compliance of pharmacists, it was demonstrated that in spite of the claim made by dermatologists regarding their compliance rate of 90% with the PPP, their compliance rate with the PPP was 25%.^50^ Furthermore, a retrospective study was carried out in Dutch between 1 January 2005 and 31 December 2008 to assess the compliance with the PPP among women of reproductive age. Comprehensive information evaluating Dutch Foundation of Pharmaceutical Statistics indicated that the use of isotretinoin increased among women in reproductive age during the study period. Furthermore, the concomitant use of oral hormonal or intrauterine contraceptives and isotretinoin was low (59.3%, 95% CI: 57.6, 61.0). Also, prescription of isotretinoin by a specialist was performed in only 78.2% of the participants. In addition, only 70% of them were treated with conventional acne therapy prior to isotretinoin initiation.^35^ Furthermore, a total of 500000 women were included in a drug utilization study in the Netherlands. Data of a drug prescription database from 1999 until 2006 to compare contraceptive use in isotretinoin and non-isotretinoin users was carried out. It was demonstrated that approximately 53% of the 651 patients on isotretinoin therapy were prescribed contraceptives before, during, and after discontinuation of isotretinoin in accordance with the PPP in comparison with approximately 43% in non-isotretinoin using group.^51^

A study was carried out in 2009 to collect data on performance of the coordinated PPP of isotretinoin in the Europe Union (EU) member states and Norway and Iceland. A questionnaire was sent to all of them. A total of 143 cases of isotretinoin exposures during pregnancy period were reported in 16 of 22 collaborating countries since implementation of the PPP. The PPP implementation was in force from 21th countries (Table 1).^52^

This review was conducted to evaluate isotretinoin use all over the world with a special consideration on pregnancy. This study demonstrated an increase in isotretinoin use with an improper prescription pattern.

Isotretinoin exposure during pregnancy and its consequent fetal abnormality in developed countries such as Canada, Germany, Netherlands and France is a concern.^45-49^ Furthermore, a questioner based study indicated same results in the EU members, Norway and Iceland.^52^

A French study reported 127 prescriptions of isotretinoin to pregnant women by dermatologists.^6^ Furthermore, it was shown that in spite of 93% of dermatologists’ claim about their compliance with the PPP, there was only a compliance rate of 25% with the pooled program in Netherland in 2011.^50^ In addition, according to a retrospective cohort study in Dutch, 30% of specialists didn’t prescribe conventional acne therapy prior to isotretinoin initiation.^35^ The data from a systematic review in Europe demonstrated a lower compliance rate of 6–26% with the PPP.^46^

**Table 1:** The summary of studies about use of isotretinoin during pregnancy

**References**	**Study design**	**Country**	**Total number of patients**	**Results**
Entezari-Maleki T, et al.^26^ 2012	Prospective, drug utilization evaluation	Iran	274	6.8% of patients used two methods of contraceptive 51.3% received isotretinoin under the usual recommended daily doses
Teichert M, et al.^35^ 2010	Retrospective study	Dutch	4881	Concomitant use of oral hormonal or intrauterine contraceptives and isotretinoin was 59.3%.
Pinheiro SP, et al.^43^ 2013	Prospective comparative study	USA	113 578	Temporary increase in concomitant use of isotretinoin and contraceptives was observed immediately after iPlEDGE implementation
Shin J, et al.^44^‏ 2011	Retrospective comparative study	USA	9912	The risk of fetal exposure after iPLEDGE implementation reduced significantly in comparison with the SMART program
Berard A, et al.^45^ 2007	Prospective population-based study	‏‍Canada	8609	A total of 9 live births occurred.
Crijns HJ, et al.^46^ 2011	Systematic review of 17 studies‏	Europe	141	0·2–1·0 per 1000 women of reproduction age receiving isotretinoin became pregnant whereas 65% – 87% of these pregnancies were terminated
Schaefer C, et al.^47^ 2010	Observational prospective study	Germany	108	A total 18 live births occurred‏
Autret-Leca E. et al.^48^ 2010	Prospective population-based study	France	147	A total of 147 cases of isotretinoin exposure were reported
Zomerdijk IM, et al.^49^ 2014	Retrospective population-based study	Netherlands	203962	Among those potentially exposed to isotretinoin, three intrauterine deaths and two live-born infants with major congenital anomalies occurred.
Crijns I, et al.^50^ 2013	Prospective descriptive study	Netherlands	556‏	In addition to poor compliance of pharmacists, that 25% of dermatologists were compliant with the PPP
Crijns HJ, et al.^51^ 2012	Prospective drug utilization study	Netherlands	651	53% of the 651 patients on isotretinoin therapy were prescribed to use contraceptive before, during, and after discontinuation of isotretinoin in accordance to the PPP.
Crijns I, et al.^52^ 2012	Prospective questionnaire- based study	Europe, Norway and Iceland	143	Exposing to isotretinoin in spite of the PPP implementation.

A study in Saudi Arabia confirmed that community pharmacists were not enough conscious about isotretinoin risks in female of childbearing potential. Furthermore, most of them didn’t recommend two methods of contraception.^42^ Furthermore, according to a prospective, drug utilization evaluation study in Iran, only 6.8% of couples used two methods of contraception.^26^ Despite a temporary successfulness, the data showed that implementation of the iPLEDGE program in the USA in order to reduce the fetal exposure was failed.

Taken together, the results of the present study showed neither of patients nor pharmacists and dermatologists were fully adherent to the risk reduction programs. In addition, exposure to isotretinoin among women during pregnancy still occurs and leads to live-born neonates with a variety of abnormalities in all over the world. 

The present mini review has some limitation. Most importantly, a total 12 revenant studies weren’t included because of unavailable free full text. Furthermore, most of included studies have been carried out in developed countries. Due to lack of risk reduction programs in low-income countries, evaluating of isotretinoin pregnancy considerations are required in these countries.

## Conclusion

The results of the presen1t study showed an increase in the use of isotretinoin. One of the important concerns is that the drug was not used with appropriate caution in pregnancy and in spite of development of the risk reduction programs, low adherence to these programs occurred and led to live births with congenital abnormalities.

## Ethical Issues

Not applicable.

## Conflict of Interest

Authors declare no conflicts of interest.
